# An updated definition of global health

**DOI:** 10.1186/s41256-025-00460-8

**Published:** 2025-10-28

**Authors:** Kathryn H. Jacobsen, Caryl E. Waggett, Olusoji Adeyi, Walter Bruchhausen, Shahanaz Chowdhury, Patricia M. Davidson, Ximena Garzón-Villalba, Lawrence O. Gostin, Liz Grant, Philip J. Landrigan, Hao Li, Mario C. Raviglione, Nancy R. Reynolds, Nelson K. Sewankambo, Brittany Seymour, Keith W. Martin

**Affiliations:** 1 Department of Health Studies, University of Richmond, Richmond, VA USA; 2 Department of Global Health Studies, Allegheny College, Meadville, PA USA; 3 Resilient Health Systems, Washington, DC USA; 4 Medical Faculty, University of Bonn, Bonn, Germany; 5 Department of Community Medicine, Bangladesh University of Health Sciences, Dhaka, Bangladesh; 6 Faculty of Medicine and Health, University of New South Wales, Sydney, NSW Australia; 7 School of Public Health and Nutrition, Universidad San Francisco de Quito, Quito, Ecuador; 8 O’Neill Institute for National and Global Health Law, Georgetown University, Washington, DC USA; 9 Centre for Global Health, University of Edinburgh, Edinburgh, Scotland, UK; 10 Global Observatory on Planetary Health, Boston College, Boston, MA USA; 11 Centre Scientifique de Monaco, Monaco, MC Monaco; 12 School of Public Health/Global Health Institute, Wuhan University, Wuhan, China; 13 Centre for Multidisciplinary Research in Health Science, University of Milan, Milan, Italy; 14 School of Nursing, Johns Hopkins University, Baltimore, MD USA; 15 School of Medicine, Makerere University, Kampala, Uganda; 16 School of Dental Medicine, Harvard University, Cambridge, MA USA; 17 Consortium of Universities for Global Health, Washington, DC USA

**Keywords:** Global health, International health, Health equity, International cooperation, Sustainable development, Climate change, Public health, Intersectoral collaboration

## Abstract

The most cited definition of global health, published in *The Lancet* in 2009, defines global health as “an area for study, research, and practice that places a priority on improving health and achieving equity in health for all people worldwide”. In this article, we propose an updated definition that expresses the motivations of diverse global health actors and makes One Health and sustainability more visible: “Global health is a field of academic study, research, policy, and applied practice that advances the equitable protection and improvement of population and planetary health”. Our “5 Ps model” illustrates global health as a grid that places health for all at the center of two axes representing four domains: (1) People, (2) Planet, (3) Priorities, and (4) Policies and Practices. The people–planet axis spans from social, economic, political, and other systems that affect human health to complex worldwide challenges such as those related to globalization, migration, pandemics, and climate change. The priorities–policies/practices axis positions global health as an action-oriented field in which factors such as human rights, international law, the global burden of disease, and evidence of economic impact inform the financing, implementation, and evaluation of multisectoral partnerships and interventions. We propose using this updated definition and the 5 Ps framework to modernize discussions of the scope and purpose of global health.

## Background

Global health was established as a distinct field in the late 1990s with the goal of replacing older, often colonial models of international health engagement with more equitable partnerships that respond to existential human threats while continuing to support improvements in population health in low- and middle-income countries. The new era of global health ushered in a broader array of funders, unprecedented resources for health, new public–private partnerships, and expanded roles for governments, businesses, and civil society organizations [1, 2]. Multilateralism and effective global governance, including greater transparency and accountability, were championed in this framing of global health, but some of the dominant actors downplayed critical questions about power imbalances, imperialism, aid dependency, and other tensions between the “Global North” and “Global South” [3].

Investment in global health surged in the early 2000s as the Millennium Development Goals (MDGs) established by the United Nations (UN) catalyzed international support for poverty reduction in 2000–2015 [4]. The documented successes of the MDG era prompted the UN General Assembly, in collaboration with UN Member States and hundreds of nongovernmental organizations, to adopt the more ambitious Sustainable Development Goals (SDGs) for 2016–2030 [5]. The SDGs were founded on the premise that narrowing the disparities between and within the world’s richest and poorest countries would yield lasting benefits for all collaborating parties while generating prosperity, preserving the planet, and fostering peace. As part of the SDG process, high-income countries made voluntary financial and technical commitments to accelerate progress on dozens of economic, health, environmental, and other targets in less-resourced countries.

The coronavirus pandemic that began in 2020 stalled progress on nearly all of the SDG targets and dampened enthusiasm for the concept of global goals even as it demonstrated the interdependence and shared vulnerabilities of all nations [6, 7]. The pandemic also revealed weaknesses in the International Health Regulations and in global governance structures and organizations, including the World Health Organization [8]. The strain on international relations during the pandemic foreshadowed the broader breakdown of global political norms in the post-pandemic years. By 2025, the United States and some other countries had begun withdrawing from participation in global governance and reframing equity, diversity, inclusion, and international cooperation as threats rather than strengths [9].

Global health has from its inception been motivated by a mix of economic considerations, humanitarian impulses, and biosecurity concerns [10]. All of these drivers of global health are currently being undermined by nationalistic and isolationistic movements that demand reduction of international aid and weaken the diplomatic cooperation required to mitigate shared threats like climate change, plastic pollution, armed conflict, and pandemics through treaties and other international instruments [11]. These rapid sociopolitical changes warrant an updated definition of global health that clarifies the scope and purpose of the field.

## Current definition

The most commonly cited definition of global health, written by Jeffrey Koplan and the executive board of the Consortium of Universities for Global Health (CUGH) and published in *The Lancet* in 2009 as a viewpoint entitled “Towards a common definition of global health” [12], defines global health as “*an area for study, research, and practice that places a priority on improving health and achieving equity in health for all people worldwide*” and explains that “global health emphasizes transnational health issues, determinants, and solutions; involves many disciplines within and beyond the health sciences and promotes interdisciplinary collaboration; and is a synthesis of population-based prevention with individual-level clinical care”. Global health differs from public health in scope, governance, and complexity, with global health initiatives targeting health issues that have been prioritized at the international or global levels rather than at local or national scales and global health decision-making involving a more diverse set of multilateral and multisectoral actors than public health [12].

The definition by Koplan et al*.* has been adopted by numerous medical, public health, and other groups, in part because it presents an ambitious vision for what the field can ultimately accomplish. However, several updates would make this definition more relevant today. Two are especially important.

First, the Koplan definition emphasizes a vision for global health transformation more than the policy-based processes that are necessary for achieving this goal. We affirm that definition’s centering of the values and ideals of global health. A commitment to ensuring that all people and all communities have an equal opportunity to achieve their own best health status motivates many individuals and groups working in the multidisciplinary, interprofessional global health space [13]. Core principles like health equity, social justice, collaborative governance, and sustainability remain central to the field [14]. However, these tenets represent only part of the rationale for investment in global health. For example, governments, corporations, and philanthropists use global health to enhance their own security, strengthen their diplomatic efforts, exercise their power, expand commercial opportunities, and elevate their reputations. The Koplan definition highlights the strengths and aspirations of global health at its best but glosses over some of the strategic and political realities that also shape this multisectoral field. A more candid and comprehensive definition must express a broader set of motivations, mechanisms, and actors involved in global health prioritization, policymaking, financing, and implementation.

Second, the Koplan definition of global health, which predates the integrated cross-sectoral vision of the SDGs, focuses exclusively on human health. Since that definition was published, climate change, biodiversity loss, persistent pollution, and other forms of environmental degradation have come to be recognized as leading threats to the health and wellbeing of current and future generations [15]. Humans are interdependent with animals and ecosystems, as emphasized in the One Health approach, and all living things depend on a healthy Earth and healthy ecosystems for survival [16]. Because environmental sustainability is now recognized as essential to safeguarding human security, an updated definition of global health must make planetary health a visible priority [17, 18].

## Proposed definition

Dozens of definitions of global health have been written in the years since the Koplan et al*.* paper was published, but that definition continues to be the most widely used one [19]. The paper presenting the definition has been cited thousands of times, and organizations in every world region feature the definition on their websites, in their institutional reports, and in their educational materials. Because this definition still holds significant weight in the field, we aim to modernize it while retaining its foundational structure. We therefore propose this update to the Koplan definition: “*Global health is a field of academic study, research, policy, and applied practice that advances the equitable protection and improvement of population and planetary health*”.

**Fig. 1:**
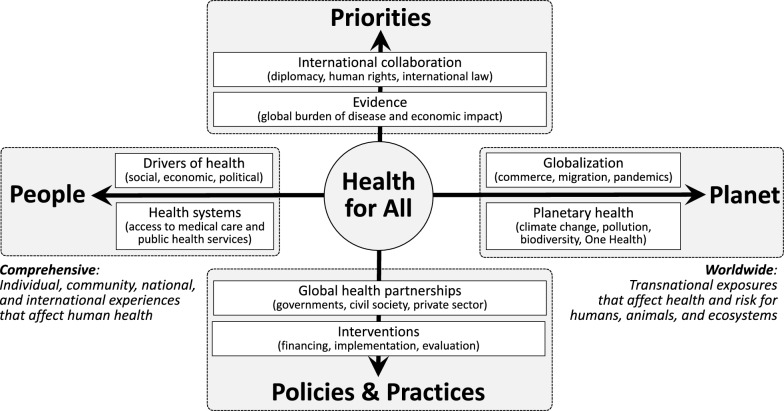
The 5 Ps model of global health

The key dimensions of a revised global health definition can be visualized using a grid that places health for all at the center of four domains: (1) people, (2) planet, (3) priorities, and (4) policies and practices. Figure 1 depicts this framework as a graph centered on health equity. The sample content provided for each domain is intended to be illustrative rather than exhaustive. Practitioners typically concentrate their work in one domain or quadrant, but the field as a whole comprises all parts of the graph [20].

Health is protected through actions that advance human rights, attenuate risk factors, and prevent new public health threats in an equitable and just manner. The people–planet axis of the 5 Ps framework identifies the many shared problems that are amenable to preventive interventions, spanning from the full set of social, economic, political, and other factors that affect access to medical care and public health services through transnational threats like pandemics and climate change that require coordinated global responses and can affect human, animal, and ecosystem health. This axis expresses both the comprehensive and worldwide meanings of the word “global” in global health. The language of protection also evokes the importance of global health security, a responsibility that has motivated government participation in many global health partnerships and initiatives.

Health is improved through actions that ameliorate existing health concerns and generate progress toward achieving health equity [21]. The priorities–policies/practices axis summarizes the full scope of activities, from upstream governance through downstream implementation, that can increase healthy lifespans, resilience, and preparedness in communities and countries around the world. These actions span from international agreements and scientific evidence that shape global health agendas to the on-the-ground activities that contribute to solving prioritized health problems. This axis applies to both academic and applied global health work, positioning global health both as a discursive and an action-oriented field.

## Implications for global health

Our proposed definition represents an incremental yet vital change for this interdisciplinary, interprofessional field. We acknowledge that this is not a transformative reimagining of global health in response to its critics, but we believe that it is necessary to clarify the current state of the field and build common ground across diverse worldviews so that stakeholders in global health can work more effectively together to shape a healthier future [22]. We have aimed to balance idealism and pragmatism in our definition, retaining health equity as the ultimate goal and framing global health as broad but not borderless. We affirm calls for continued reflection and dialogue about power dynamics and politics in global health to ensure that the field evolves in ways that are responsive to critical perspectives and changing global realities [23].

We write with a vision of strengthening, rather than abandoning, global health governance. Recognizing weaknesses in the current model of international collaboration is a critical first step toward improving the processes and structures that enable governments and their partner organizations to set effective policies, provide services, protect human rights, and build public trust. Since governments and governmental agencies may not always represent the best interests of their populations, especially in fragile states and nondemocratic settings, a model of global health that places the wellbeing of people at the center and welcomes contributions from a range of disciplines, professions, and sectors will be better equipped to meet complex health challenges now and create more effective, inclusive, transparent, accountable, and resilient systems for the future.

As leaders within CUGH and other global health organizations, we advocate for a transition from the original definition by Koplan et al*.* to this amended definition. We believe that this updated definition and the 5 Ps framework—people, planet, priorities, policies and practices—can enrich conversations about the field’s scope, relevance, and value and modernize how the field of global health is conceptualized and applied in teaching, research, and policy contexts.

## Data Availability

Not applicable.

## Abbreviations

5 Ps: People, Planet, Priorities, and Policies and Practices
CUGH: Consortium of Universities for Global Health
MDGs: Millennium Development Goals
SDGs: Sustainable Development Goals
UN: United Nations
